# Notes for Contributors

**DOI:** 10.1155/S1463924680000430

**Published:** 1980


had been unused for some time within the calibration period could give erroneously low results. This was most likely to happen if a series of urine samples were analysed followed by a serum, but the effect was by no means reproducible. The manufacturer is aware of this problem, which is probably caused by deterioration of the reagent in the measuring cup, and is taking steps to correct it.

### Creatinine

The Astra system seems to have overcome many of the problems associated with the kinetic Jaffe procedure. The method is precise and free from interference by bilirubin and haemoglobin except at very high concentrations. The interference by methyl dopa has been previously described [3].

### Summary and conclusions

With these reservations, the Astra was found to be a precise and accurate instrument for the measurement of electrolytes, urea, glucose and creatinine in serum and urine. It is easy to use, requiring only simple routine maintenance on a daily, weekly, and monthly routine. In the event of a failure of almost any part of the system, the RAPid kit enables the user to replace defective modules easily and quickly. It could comfortably analyse 200-300 specimens per day and is therefore particularly suitable for the medium-sized laboratory. It would also be suitable for use in larger laboratories to handle emergency specimens by day or night and to back up a larger multichannel analyser. It is similar in speed and capability to other instruments but has the advantage of analysing serum urea, creatinine, glucose, and electrolytes together and it will also analyse urine specimens.

ACKNOWLEDGEMENTS

The authors are indebted to Beckman RIIC Ltd for making the Astra-8 available for this evaluation. The assistance of Mr G Greaves and Mr R Bunce, who inspected the electrical and mechanical aspects of the machine is acknowledged, and also that of Mrs M Peters, Mr R Holder and Mr J Parekh for their help with the analysis of the results. The financial assistance of the Department of Health and Social Security is also acknowledged.

REFERENCES

[1] Broughton, P.M.G., Gowenlock, A.H., McCormack, J.J. and Neill, D.W., *Annals of Clinical Biochemistry,* 1974, **11**, 207-218.

[2] Lloyd, P.H., Annals of Clinical Biochemistry, 1978, **15**, 136-145.

[3] Finley, P.R., Williams, R.J., Lichti, D.A. and Thies, A.C., Clinical Chemistry 1978, **24**, 2125-2131.

[4] Truchaud, A., Hersant, J., Glikmanas, G. and Fievet, P., Abstracts of Papers and Posters 3rd European Congress of Clinical Chemistry, 1979, Brighton, p 44.

[5] North, J.W., Chittenden, C.G. and Macdonald, S.A. Abstracts of Papers and Posters 3rd European Congress of Clinical Chemistry, 1979, Brighton, p 44.

[6] Fievet, P. and Truchaud, A., Abstracts of Papers and Posters 3rd European Congress of Clinical Chemistry, 1979, Brighton, p 44.

[7] Finley, P.R. and Williams, R.J. Abstracts of Papers and Posters 3rd European Congress of Clinical Chemistry, 1979, Brighton, p 44.

### Notes for Contributors

### Presentation of manuscripts

Manuscripts should be typed (double-spaced) on one side of the paper only and with generous margins. The title should be brief and informative avoiding the word "new" and its synonyms. The full list of authors with their affiliations and full address(es) should appear on the title page. On a separate sheet an abstract of no more than 150 words is required. This should succinctly describe the scope of the contribution and highlight significant findings or innovations. It should be written in a style which can easily be translated into French and German.

*The Concise Oxford Dictionary* and Fowler's *Modern English Usage* (both published by Oxford University Press) should be used as the standard for spelling and grammar. Abbreviations should be limited to those generally recognised, or where a frequently occuring term is abbreviated it should, in the first instance, be explained thus "flow injection analysis (FIA) ..." and the abbreviation used thereafter. Abbreviations, for standard measures and units should follow SI recommendations. There are various publications giving guidance on the use of SI units.

References should be indicated in the text by numerals following the author's name, i.e. Skeggs [6]. On a separate sheet of paper, list all references in numerical order thus:

[6] Skeggs, L.T., *American Journal of Clinical Pathology,* 1959, **28**, 311.

Note that journal titles are given in full. Where there is more than one author, the form Foreman et al. should be used in the text but all authors should be named in the list of references. When reference is made to a chapter in a book the reference should take the following form:

[7] Malmstadt, H.V. in "Topics in Automatic Chemistry" Ed. Stockwell P.B. and Foreman J.K. 1978 Horwood, Chichester, pp. 68-70.

Only work which has been published or has been accepted for publication should be cited. Avoid the citation of documents which are subject to restricted circulation, patent literature, unpublished work and personal communications. The latter can be mentioned in the text in parenthesis.

To illustrate a paper line diagrams are preferred to photographs. Photographs should only be used when they significantly add to the discussion. Diagrams, charts and graphs should be carefully drawn in black ink on stout card or heavy quality tracing paper. Most illustrations are reduced for publication; to allow for this originals should be between 16 and 36 cm wide (the depth must not exceed 50 cm). The lettering of diagrams should be sufficiently clear to withstand reduction. Except in the case of proper names, all lettering should be in lower case print. If photographs are used they must be supplied in the form of clear, unmounted, glossy, black and white prints. "Instant" photographs are not normally acceptable. All illustrations must be identified on the reverse showing the figure number and the author's name.

Each illustration should have a fully explanitory caption. Captions should be typed together on a separate sheet of paper; they must *not* be an inseparable part of the illustration.

